# 3D-printed mesoporous bioactive glass/GelMA biomimetic scaffolds for osteogenic/cementogenic differentiation of periodontal ligament cells

**DOI:** 10.3389/fbioe.2022.950970

**Published:** 2022-10-18

**Authors:** Nianrou Mei, Yiwen Wu, Binglin Chen, Tian Zhuang, Xinge Yu, Baiyan Sui, Tingting Ding, Xin Liu

**Affiliations:** ^1^ Department of Dental Materials, Shanghai Biomaterials Research & Testing Center, Shanghai Ninth People’s Hospital, Shanghai Jiao Tong University School of Medicine, College of Stomatology, Shanghai Jiao Tong University, National Center for Stomatology, National Clinical Research Center for Oral Diseases, Shanghai Key Laboratory of Stomatology, Shanghai, China; ^2^ School and Hospital of Stomatology, Tongji University, Shanghai Engineering Research Center of Tooth Restoration and Regeneration, Shanghai, China; ^3^ Department of Oral and Cranio-maxillofacial Science, Shanghai Ninth People’s Hospital, Shanghai Jiao Tong University School of Medicine, College of Stomatology, Shanghai Jiao Tong University, National Center for Stomatology, National Clinical Research Center for Oral Diseases, Shanghai Key Laboratory of Stomatology, Shanghai, China

**Keywords:** biomimetic scaffolds, 3D printing, bioactive glass, GelMA, periodontal regeneration

## Abstract

Integrated regeneration of periodontal tissues remains a challenge in current clinical applications. Due to the tunable physical characteristics and the precise control of the scaffold microarchitecture, three-dimensionally (3D) printed gelatin methacryloyl (GelMA)-based scaffold has emerged as a promising strategy for periodontal tissue regeneration. However, the optimization of the printing biomaterial links the formulation and the relationship between the composition and structures of the printed scaffolds and their comprehensive properties (e.g. mechanical strength, degradation, and biological behaviors) remains unclear. Here, in this work, a novel mesoporous bioactive glass (BG)/GelMA biomimetic scaffold with a large pore size (∼300 μm) was developed by extrusion-based 3D printing. Our results showed that the incorporation of mesoporous bioactive glass nanoparticles (BG NPs) significantly improved shape fidelity, surface roughness, and bioactivity of 3D-printed macroporous GelMA scaffolds, resulting in the enhanced effects on cell attachment and promoting osteogenic/cementogenic differentiation in human periodontal ligament cells. The excellent maintenance of the macropore structure, the visibly improved cells spreading, the release of bioactive ions (Si^4+^, Ca^2+^), the upregulation of gene expressions of osteogenesis and cementogensis, and the increase in alkaline phosphatase (ALP) activity and calcium nodules suggested that BG NPs could endow GelMA-based scaffolds with excellent structural stability and the ability to promote osteogenic/cementogenic differentiation. Our findings demonstrated the great potential of the newly formulated biomaterial inks and biomimetic BG/GelMA scaffolds for being used in periodontal tissue regeneration and provide important insights into the understanding of cell–scaffold interaction in promoting the regeneration of functional periodontal tissues.

## 1 Introduction

The average worldwide prevalence of severe periodontitis has been estimated to be 11%, including countries with relatively less emphasis on periodontal health care (Slots, 2017). For patients with severe periodontitis, the loss of periodontal bone tissue is irreversible. Current clinical approaches can only control the progression of periodontal disease and set back the destruction of the periodontal tissue, but can hardly regenerate the periodontal tissue, especially for those teeth with deep pockets. Up until now, severe periodontitis associated with deep intrabony defects is considered as a clinical challenge (Cortellini and Tonetti, 2015). As is well known, the key to overcoming the challenge is the tissue-engineering strategy, which creates replacement tissues using a combination of cells, scaffolds, and growth factors and promotes tissue regeneration (Iwata et al., 2014). However, owing to the complicated biological evaluation and the high cost of synthesis, the clinical translation of tissue-engineering products with seeding exogenous stem cells remains quite limited (Gao et al., 2021). Thus, it is of great importance to design and develop desirable cell-free scaffolds to stimulate endogenous regeneration for repairing periodontal soft/hard tissue defects.

To achieve successful tissue regeneration, implanted scaffolds need to have the capability to recapitulate the structural and compositional aspects of the tissue, which important for restoring tissue function (Jeon et al., 2014). Depending on the use of a singular homogeneous biomaterial or biphasic or multiphase heterogeneous biomaterials, scaffolds for tissue regeneration can be divided into single-phase, dual-phase, and multiphase (Deliormanlı and Atmaca, 2018). Due to the limitation of its structure and composition, single-phase scaffolds cannot meet the need for the simultaneous regeneration of multiple tissues (Gong Jinglei and Wang, 2021), while multiphase scaffolds can be composed of diverse materials or loaded with a variety of bioactive molecules to mimic the complex tissue structure or promote multiple tissue regeneration. In the repair of osteochondral defects, scaffold–cell constructs were specifically designed to mimic the physiological properties and structure of two different tissues (cartilage and bone) (Yousefi et al., 2015). This concept can be applied to periodontal tissue regeneration. For example, a multiphase polycaprolactone/hydroxyapatite scaffold with different pore/channel scales was fabricated using three-dimensional (3D) printing and was found to regenerate a periodontium complex by time-releasing various functional proteins such as amelogenin, connective tissue-growth factor, and bone morphogenetic protein-2 (Lee et al., 2014). Also, a porous tri-layered nanocomposite hydrogel scaffold composed of chitin-poly (Sowmya et al., 2017a) and nano-bioactive glass–ceramic with various functional proteins (i. g. cementum protein 1, fibroblast growth factor 2, and platelet-rich plasma-derived growth factors) was reported to promote cementogenic, fibrogenic, and osteogenic differentiations of human dental follicle stem cells (Sowmya et al., 2017b). However, in these studies, the integrated multiple-tissue regeneration largely depended on the delivery of various growth factors which would limit the long-term benefits and clinical translation because of their short effective half-life, low stability, and rapid inactivation under physiological conditions (Wang et al., 2017).

**SCHEME 1:**
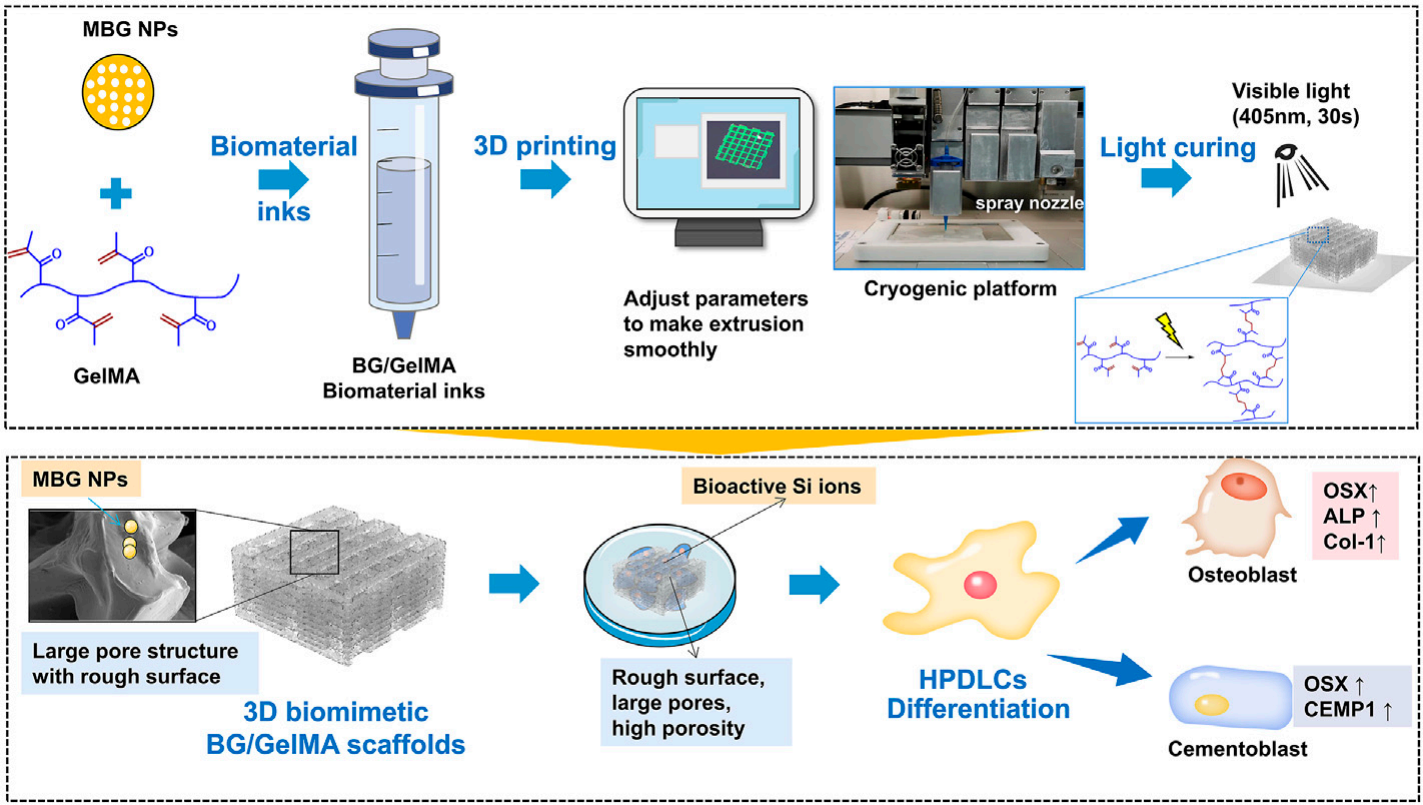
Schematic elucidating the 3D printing strategy of biomimetic scaffolds with BG/GelMA inks for osteogenic and cementogemic differentiation of human periodontal ligament cells (HPDLCs). Mesoporous bioactive glass nanoparticles: MBG NPs; Gelatin methacryloyl: (GelMA).

Among manufacturing technologies for scaffolds, 3D printing enabled precise control of the scaffold’s microarchitecture, showing a promising prospect in realizing simultaneous integrated periodontal-tissue regeneration. Generally, 3D printing has a variety of methodologies, such as inkjet printing, laser-assisted 3D printing, extrusion, and so on which are suitable for various biomaterials and printing needs (Tao et al., 2019). In recent years, 3D-printed hydrogel-based scaffolds have attracted increasing attention for bone and cartilage-tissue regeneration because they can mimic the 3D microenvironment of the native extracellular matrix, provide a porous channel-rich structure to supply nutrients for cell growth and differentiation, and offer tunable geometric shapes to repair irregular bone defect (Gao et al., 2019; Luo et al., 2022). For 3D-printed hydrogel-based scaffolds, one of the most commonly used natural bioink is gelatin methacryloyl (GelMA), which is derived from a hydrolytic degradation of collagen (Janmaleki et al., 2020.). Currently, GelMA hydrogels have been widely used for various biomedical applications due to their suitable biological properties and tunable physical characteristics (Yue et al., 2015; Kamkar et al., 2021). It has been reported that GelMA hydrogels closely resemble some essential properties of native extracellular matrix (ECM) which allows cells to proliferate and spread in GelMA-based scaffolds (Alge and Anseth, 2013; Yue et al., 2015). Light crosslinking makes GelMA from liquid to gel with increasing mechanical strength and stability. Even so, applications of GelMA hydrogels are still limited due to the low mechanical strength and poor printability (Xiao et al., 2019). Furthermore, the pore size of scaffolds is also a critical parameter that modulates cell biological behaviors including osteogenesis, chondrogenesis, and vascularization. It has been reported that scaffolds with large macropores (greater than 250 μm in diameter) facilitate the osteogenic differentiation of bone marrow mesenchymal stem cells and robust vascularization (Swanson et al., 2021). Thus, numerous efforts are devoted for improving the mechanical property and bioactivity of 3D-printed GelMA scaffolds by incorporating various inorganic nanomaterials (e.g. nanoclay, silica nanoparticles, hydroxyapatite nanoparticles, etc.) (Gao et al., 2021; Tavares et al., 2021). However, such 3D-printed GelMA multiphase scaffolds still present some challenges regarding the optimization of the printing biomaterial ink formulation and macropore structure. Moreover, the relationship between the composition and structures of the printed scaffolds and their comprehensive properties (e.g. mechanical strength, degradation, and biological behaviors) remains unclear. Among the most popular bioactive inorganic nanoparticles, bioactive glass nanoparticles (BG NPs), mainly comprised of SiO_2_, CaO, and Na_2_O, have been highlighted in bone regeneration due to their excellent osteoinductive capability, which can lead to the formation of a hydroxyapatite layer with a bond forming between the tissue and the material (Skallevold et al., 2019). However, bioactive glass has several limitations such as the difficulty of being processed into 3D scaffolds and a low-degradation rate which hardly matches the formation rate of new tissue (Rahaman et al., 2011). Herein, in this work, to mimic the organic component and 3D microenvironment of native ECM in the periodontal tissue, both gelatin and mesoporous BG NPs were selected for the biomaterial ink formulation due to gelatin derived from type I collagen and the osteoinductive activity of bioactive glass, and then a novel biomimetic BG/GelMA macroporous scaffold with a large pore size (∼300 μm) was developed by 3D printing through controlling the amounts of BG NPs. With increasing mesoporous BG NPs incorporation, the 3D-printed GelMA hydrogel scaffold displayed higher structural stability, rougher surface, and better bioactivity, which is more suitable for cell attachment, spreading, and osteogenic/cementogenic differentiation in periodontal ligament cells (Scheme 1). The 3D-printed biomimetic bioactive glass/GelMA macroporous scaffolds show good shape fidelity, biocompatibility, and excellent osteogenesis/cementogensis ability, suggesting a promising material for integrated periodontal tissue regeneration.

## 2 Materials and methods

### 2.1 Synthesis and characterization of mesoporous BG NPs

Mesoporous BG NPs used in this study were prepared using a cetyl pyridine bromide (CPB) template method according to the previously published protocol (Sui et al., 2018). Briefly, 0.23 g NaOH and 1.0 g of polyvinylpyrrolidone (PVP) were dissolved in 120 ml ddH_2_O. After stirring for 10 min, 1.4 g of CPB (Sigma-Aldrich, St. Louis, United States) was dissolved in the solution and stirred continuously for one hour. Next, tetraethyl orthosilicate (TEOS), calcium nitrate tetrahydrate, and TEP (the molar ratio of Ca: P: Si = 15: 5: 80) were subsequently added and stirred for 24 h. The solution was collected and washed with ddH_2_O three times and then sealed in Teflon-lined autoclaves at 80°C for 48 h. Finally, the dispersion was dried at 80°C for 12 h and calcined at 550°C for five hours to obtain mesoporous BG NPs.

The structure and morphology of the BG NP sample were characterized by high-resolution transmission electron microscopy (TEM, JEM-2100, JEOL), and the size distribution was calculated by using ImageJ analysis software (Media Cybernetics Inc., United States). The Brunauer–Emmett–Teller (BET) specific surface area and pore size distribution of MBG were determined using a micromeritics porosimeter (ASAP 2460, Micrometrics Instrument).

### 2.2 Preparation of biomaterial inks

Gelatin methacryloyl (GelMA) and photoinitiator LAP (phenyl-2,4,6-trimethylbenzoylphosphi-nate) were purchased from Suzhou Intelligent Manufacturing Research Institute (Suzhou, China). First, 20 mL phosphate buffer saline (PBS, HyClone, Logan, UT, United States) was added into a brown flask containing 0.05 g LAP powder to obtain 0.25% (w/v) LAP solution, and then it was sterilized by a syringe filter with a pore size of 0.22 μm (Merck Millipore, Billerica, MA, United States). Subsequently, as shown in Table 1, different ratios of BG NPs and 10% (w/v) of GelMA powder were added into four LAP solutions to prepare the biomaterial inks for the 3D-printed scaffolds, melted in a water bath (60°C, 30 min), and oscillated three times. The four kinds of biomaterial inks were named as 0% BG, 1% BG, 5% BG, and 10% BG with respect to the weight of GelMA (w/w).

**TABLE 1:** The component of each group of biomaterial inks and scaffolds.

Biomaterial inks	Scaffolds	PBS (ml)	LAP(g)	GelMA (g)	BG NPs (g)
Ⅰ	0% BG	20	0.05	2.0	0.00
Ⅱ	1% BG	20	0.05	2.0	0.02
Ⅲ	5% BG	20	0.05	2.0	0.10
Ⅳ	10% BG	20	0.05	2.0	0.20

PBS, phosphate buffer saline; GelMA, gelatin methacryloyl; BG, bioactive glass; LAP, Lithium phenyl-2,4,6-trimethylbenzoylphosphinate.

### 2.3 Rheological characterization of biomaterial inks

Rheological properties of the biomaterial inks were performed by using a rheometer (Anton Paar MCR302, Austria) equipped with parallel plates with a diameter of 25 mm and a truncation gap distance of 2.6 mm. The linear viscoelastic range (LVR) was obtained from the single frequency amplitude sweep. To measure the viscosity, these inks were loaded with steady-rate sweeps within a shear rate range of 0.01–1,000 s^−1^. To measure the storage modulus (G′) and loss modulus (G″), frequency sweep tests were conducted in the linear viscoelastic region at a strain of 1.0% according to previously reported methods (Gao et al., 2019; Schwab et al., 2020b). All experiments were performed in room temperature (RT = 25°C). Before the experiments, the biomaterial inks were cooled to 4 °C for 30 min to get the transformation from liquid to gel.

### 2.4 Preparation of biomimetic BG/GelMA scaffolds

The biomimetic BG/GelMA scaffolds were constructed by an extrusion-based 3D printer (Motor Assisted Microsyringe, Shanghai fuqifan Electromechanical Technology Co., Ltd., Shanghai, China). First of all, a parameter file was designed. The scaffold was designed as a 1×1 × 0.5 cm (long×wide×high) rectangle. The filament diameter was 400 μm and the filament space was 1.2 mm. The temperature of the bin and print plate were 20–25°C and 15°C, respectively. The moving speed of the nozzle (inner diameter: 400 μm) was 10 mm/s and the extrusion pressure was 0.3–0.6 MPa. Then, the photocurable crosslinked biomaterial inks were loaded into the printer and the program was started. During the printing process, the external computer transmitted the 3D model data of the scaffolds to the control system of the printer and drove the printing nozzle to move along the X–Y directions so that the ink could be stacked into the section of the model. After the printing of the section was completed, the nozzle was raised to the next section layer, and the printing process was repeated until the supported printing was completed. After printing, crosslinking curing was carried out under UV-lamp irradiation. The wavelength of the UV light was 405nm, the illumination intensity was 25 mW/cm^2^, and the irradiation time was 50 s. The scaffolds were refrigerated in PBS and the scaffolds prepared with printing biomaterial inks of 0% BG, 1% BG, 5% BG, and 10% BG were labeled as scaffolds 0% BG, 1% BG, 5% BG, and 10% BG, respectively.

### 2.5 Morphological characterization of biomimetic BG/GelMA scaffolds

The morphological structure of the 3D-printed BG/GelMA scaffolds was observed from macro and micro perspectives. For gross observation, the length and height of the scaffolds were measured with a ruler and determined according to images taken by a camera. From a micro perspective, the morphology of scaffolds including the pore structure was visualized using scanning electron microscopy (SEM, Tescan Mira 3 XH, Czech Republic) and the incorporated mesoporous BG NPs were characterized by using an energy dispersive spectrometer (EDS, AZtec X-Max^N^ 80, United Kingdom).

The pore size and porosity were measured and calculated using the threshold method by using ImageJ (Media Cybernetics Inc., United States) analysis software (Grove and Jerram, 2011; Loh and Choong, 2013). Specifically, SEM images of the scaffolds were opened, the scale was adjusted, the image was cropped to make a rectangular section only comprising of the sample, it was converted to an 8-bit paletted file, the threshold tool was used to select the porous areas, and the particles tool was analyzed to get the pores’ diameter. The displayed results were copied and analyzed in Excel (Microsoft, China) and Prism 9 (GraphPad Software, LLC. United States), respectively.

### 2.6 Thermal behavior and infrared analysis of biomimetic BG/GelMA scaffolds

The thermal stability of scaffolds was studied through thermogravimetric analysis (Netzsch STA 449 F3 Jupiter®, Germany) from room temperature to 700°C at a heating rate of 10°C/min under Ar flow.

The chemical compositions of the biomimetic BG/GelMA scaffolds were assayed by using (FTIR) Fourier transform infrared spectroscopy with KBr powder on a DTGS spectrometer (Thermo Scientific, United States). The samples were dried in constant temperature under 40 °C in an oven and crushed down. Spectra were recorded in the range of 500–4,000 cm^−1^ at 32 scans with a resolution of 4 cm^−1^.

### 2.7 Mechanical property of biomimetic BG/GelMA scaffolds

The mechanical property of the 3D-printed BG/GelMA scaffolds was measured by performing a compression test using a universal testing system loaded on a microcomputer-controlled electronic universal material-testing machine (Shanghai Hengyi Precision Instrument Co., LTD, China) at a speed of 1 mm/min until above 90% deformation of the sample. The sample scaffolds were 10.0 mm×10.0 mm×5.0 mm (long × wide × height) with lattice structure (7 × 7, meaning 7 columns and 7 rows) with 0◦, 90◦ stacking (12 layers). The compressive modulus was calculated by the slope of the linear region of the stress–strain curve, almost limited to 5%–20% of the strain. All samples were tested thrice.

### 2.8 Degradation property of biomimetic BG/GelMA scaffolds

The degradation property of the 3D-printed BG/GelMA scaffolds was evaluated using a gravimetric method. Firstly, the original weight M0 of all test samples was recorded. Secondly, under sterile operation, the test sample (N = 3) was completely immersed in PBS at a ratio of 1g: 30 ml within a sealed container and placed in a 37°C oven. At various time-points (1 day, 3 days, 7 days, 14 days, and 28 days), the test sample was weighted and recorded as M1. The degradation ratio was assessed by monitoring the weight changes of the scaffolds using the following equation:

Degradation ratio(%)=(M0−M1)/M0×100%

Moreover, the scaffolds were submerged in a solution of 1 mg/ml collagenase 2 solutions (ThermoFisher, Waltham, MA, United States) at a ratio of 1g: 10 ml. The degradation was carried out at 37°C and 100rpm in an oscillator. The original weight M0 of all test samples was recorded. The scaffolds were observed every 15 min and their wet weight was recorded as M2 at that time. The mass loss ratio was assessed by monitoring the weight changes of the scaffolds using the following equation:

Mass loss ratio (%)=(M0−M2)/M0×100%

### 2.9 Cell morphology on the surface of biomimetic BG/GelMA scaffolds

Human periodontal ligament cells (HPDLCs) were purchased from ScienCell Research Laboratories, Inc. (catalog #2630, Carlsbad, United States) and cultured in a cell medium (ScienCell, Carlsbad, United States) containing 2% fetal bovine serum (FBS, ScienCell, Carlsbad, United States), 1% penicillin/streptomycin solution (P/S, ScienCell, United States), and 1% growth supplement (ScienCell, Carlsbad, United States) in a humidified atmosphere of 5% CO_2_ at 37°C. In detail, the BG/GelMA scaffolds were sterilized by ultraviolet light for 4 h and placed in a 24-well cell-culture plate. Subsequently, HPDLCs at a density of 2.0 × 10^5^/ml were seeded on the surface of as-prepared scaffolds for 24h and then fixed in 2.5% glutaraldehyde (4°C) overnight. The cell morphology, adhesion, and growth on the surface of the scaffolds were observed by SEM (Tescan Mira 3 XH, Czech Republic).

### 2.10 Cell viability assay

The CellTiter 96 ® AQueous one solution assay (Promega, Madison, WI, United States) was used to evaluate the cell viability of HPDLCs cultured in the as-prepared BA/GelMA scaffolds for 3 days. Then, the HPDLCs cultured with different scaffolds were incubated with a cell medium containing 20% v/v of MTS (3-(4,5-dimethylthiazol-2-yl)-5-(3carboxymethoxyphenyl)-2-(4-sulfophenyl)-2H-tetrazolium). After 4 h, the supernatants were transferred to new 96-well plates and the absorbance at 490 nm was determined on a microplate reader (Multiskan GO, Thermo Scientific, MA, United States).

### 2.11 Inductively coupled plasma mass spectrometry (ICP-MS) analysis

To investigate whether the ion-release of biomimetic BG/GelMA scaffolds could promote osteogenic/cementogenic differentiation of HPDLCs, cells were cultured in the scaffolds 0% BG, 1% BG, 5% BG, and 10% BG for day 1 and day 7, respectively. Subsequently, the concentrations of Ca, Si, and P ions in the cell-culture medium extracts of as-prepared scaffolds were respectively measured by inductively coupled plasma mass spectrometry (ICP-MS) (Agilent7700s, Agilent Technologies Co. Ltd, United States). The number of replicates used in this experiment was three.

### 2.12 Alkaline phosphatase staining

ALP staining was performed using BCIP/NBT Alkaline Phosphatase Color Development Kit (Beyotime Biotechnology, Shanghai, China) to evaluate the osteogenesis differentiation of HPDLCs cultured in the as-prepared BG/GelMA scaffolds. In detail, HPDLCs were cultured in the scaffolds 0% BG, 1% BG, 5% BG, and 10% BG for day 7, 14, and 21, respectively. According to the requirements of the ALP kit, added 5 ml ALP color-developing buffer and 16.5 μl BCIP solution (300X), NBT solution (150x) 33 μL, and BCIP/NBT solution 5.05 ml into the test tube, in turn, was mixed to prepare BCIP/NBT dyeing working solution. After washing the scaffold samples (on day 7, 14, 21) with PBS, the washing solution was removed and added BCIP/NBT dyeing working solution to ensure that the samples can be fully covered, and then the scaffold samples were incubated in the dark for 5–30 min until the color developed to the expected depth. We removed the BCIP/NBT dyeing working solution, washed it with distilled water 1–2 times, stopped the color reaction, and the results of each group were observed and recorded.

### 2.13 Alizarin Red S staining

Alizarin Red S staining (Beyotime Biotechnology, Shanghai, China) was used to observe extracellular matrix calcification of HPDLCs cultured in the as-prepared BG/GelMA scaffolds. HPDLCs were inoculated with the scaffolds of each group for 21 days, washed with PBS, removed from the washing solution, added to the fixed solution for 20 min, and washed 3 times by PBS. According to the requirements of the kit, we added an appropriate amount of Alizarin Red S dyeing working solution to ensure that the sample can be fully covered, dyed at room temperature for 30 min, and the results of each group were observed after fully washing with distilled water. The results of each group were observed and recorded.

### 2.14 Real-time quantitative RT-PCR

The osteoblast/cementoblast-related gene transcription of Osterix (OSX), Cementum protein-1 (CEMP-1), ALP and type I collagen (Col-1a1) was detected by real-time quantitative reverse transcriptase–polymerase chain reaction (qRT-PCR). Briefly, HPDLCs were cultured in the scaffolds 0% BG, 1% BG, 5% BG, and 10% BG for 7 days, and the total RNA of HPDLCs was extracted using the RNeasy® Mini kit (QIAGEN, Germany). Complementary DNA (cDNA) was synthesized from the total RNA using the PrimeScript RT Reagent Kit (TaKaRa, Japan) according to the manufacturer’s instructions. qPCR was performed by using a LightCycler® System (Roche Diagnostics, United States) using TB Green Premix Ex Taq II (Tli RNaseH Plus) (TaKaRa, Japan). The housekeeping gene GAPDH was used to normalize the results. The primer sequences used in this study are listed in Table 2.

**TABLE 2:** Primers used for real-time quantitative RT-PCR.

Target gene	Primer sequence	
	Forward	Reverse
OSX	CCT​CTG​CGG​GAC​TCA​ACA​AC	AGC​CCA​TTA​GTG​CTT​GTA​AAG​G
ALP	ACC​ACC​ACG​AGA​GTG​AAC​CA	CGT​TGT​CTG​AGT​ACC​AGT​CCC
Col-1α1	GTG​CGA​TGA​CGT​GAT​CTG​TGA	CGG​TGG​TTT​CTT​GGT​CGG​T
CEMP-1	GGG​CAC​ATC​AAG​CAC​TGA​CAG	CCC​TTA​GGA​AGT​GGC​TGT​CCA​G
GAPDH	ACA​ACT​TTG​GTA​TCG​TGG​AAG​G	GCC​ATC​ACG​CCA​CAG​TTT​C

### 2.15 Statistical analysis

All data are presented as the mean ± standard deviation. Statistical analysis was performed using the Prism 9 statistical software package (GraphPad Software, LLC. United States). An ordinary one-way analysis of variance (ANOVA) was performed, followed by Turkey’s post hoc test to determine the differences between the scaffolds 0% BG, 1% BG, 5% BG, and 10% BG. In all cases, significance was asserted at *p* < 0.05.

## 3 Results

### 3.1 Characterization of the biomaterial BG/GelMA inks

#### 3.1.1 Characterization of mesoporous bioactive glass nanoparticles

**FIGURE 1:**
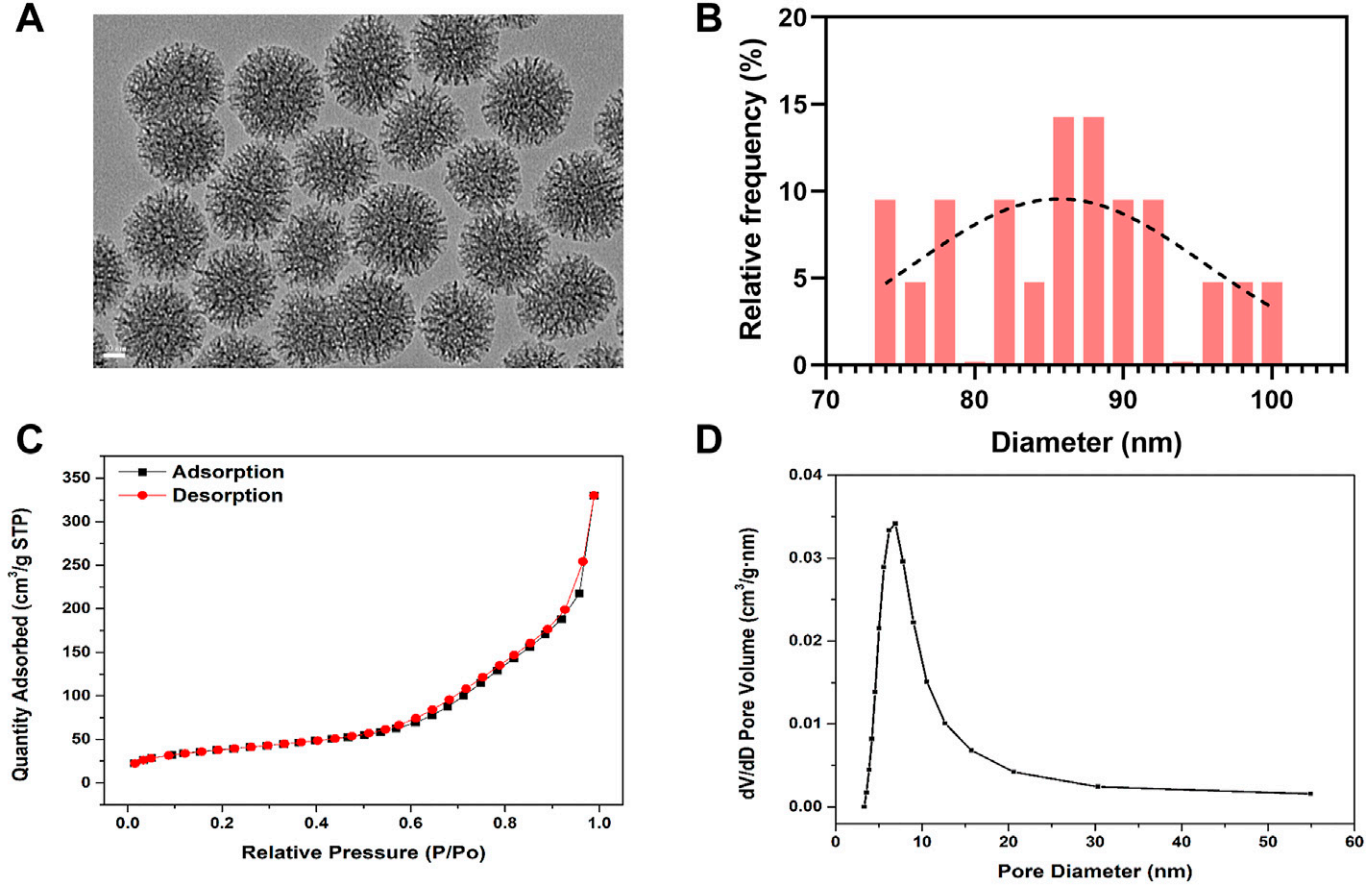
Characterization of mesoporous bioactive glass nanoparticles (BG NPs). **(A)** TEM images. Scale = 20 nm. **(B)** Particle-size analysis by ImageJ of mesoporous BG NPs. **(C)** Nitrogen adsorption–desorption isotherms and **(D)** Pore-size distribution of BG NPs.

The morphology of BG NPs was characterized by transmission electron microscopy (van Haaften et al., 2019) (Figure 1A). BG NPs exhibits an obvious multi-generational hierarchical dendritic structure: inner mesoporous BG NPs exhibit hexagonal channels and external mesoporous BG NPs show radial channels, the mean particle size was measured as 85.97±7.60 nm (Figure 1B). We further confirmed the mesoporous structure of BG NPs by using the nitrogen sorption analysis. Figure 1C shows the N_2_ adsorption–desorption of type IV isotherm and pore-size distribution for mesoporous BG NPs, which is a typical isotherm for mesoporous materials. Specifically, the specific surface area, pore volume, and pore size of mesoporous BG NPs are 134.84 m^2^/g, 0.51 cm³/g and 9.10 nm, respectively (Figure 1C,D).

#### 3.1.2 Rheological properties of the biomaterial inks

**FIGURE 2:**
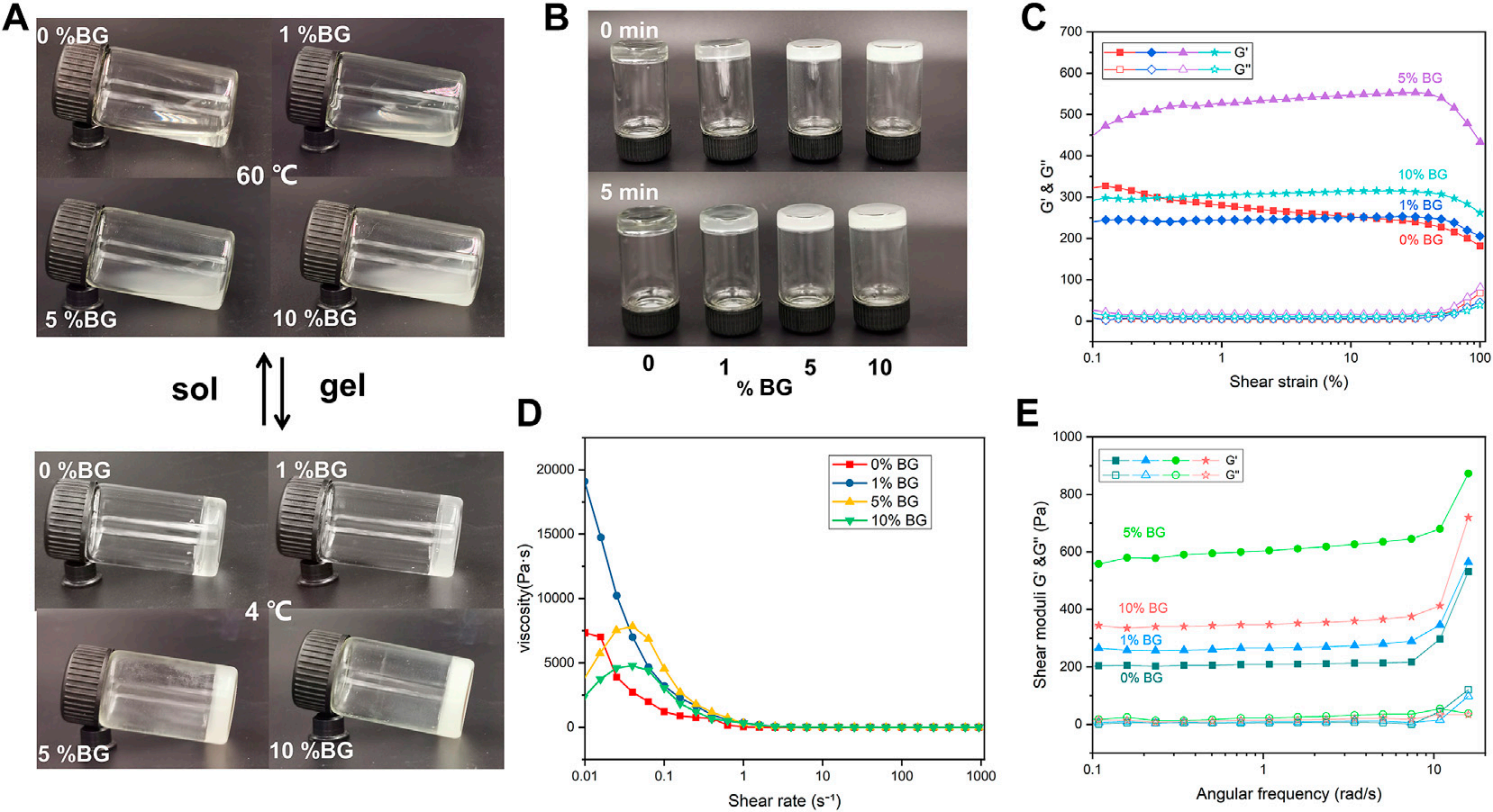
Rheological properties of the BG/GelMA biomaterial ink: flow behavior of 0%, 1%, 5%, and 10% BG **(A)** at 60 °C and 4 °C **(B)** after 0 min and 5 min **(C)** the elastic moduli (G′) and viscous moduli (G″) shear–strain; **(D)** the viscosity–shear rate; and **(E)** the shear moduli–angular frequency of the respective biomaterial inks. The ‘sol’ means the biomaterial inks went from liquid to gel and the ‘gel’ means from gel to liquid.

To assess the printability of the biomaterial inks, rheological properties including viscosity, viscoelasticity, and shear-thinning were measured. As shown in Figures 2A,B the biomaterials inks (0% BG, 1% BG, 5% BG, and 10% BG) showed different flow behaviors. Viscosity is the resistance of a fluid to flow. It has been observed that the viscosity of the 10% BG group is higher than that of the 0% BG group. Furthermore, the viscoelasticities of four biomaterial inks were measured within the linear viscoelastic region (LVR) *via* shear–strain sweeps, which can be described by the viscous components (storage modulus G′) and the elastic components (loss modulus G″). Figure 2C shows that the GelMA inks containing 1%–10% BG NPs have clearer linear-viscoelastic range (LVR), compared with the GelMA inks without BG NPs. Figure 2D shows that all the biomaterial inks have shear-thinning behavior with increasing shear rate, and the storage modulus of the GelMA inks containing BG NPs is higher than the loss modulus (Figure 2E), suggesting that BG NP incorporation could increase the stability of the GelMA inks.

### 3.2 Synthesis and characterization of the biomimetic BG/GelMA scaffolds

**FIGURE 3:**
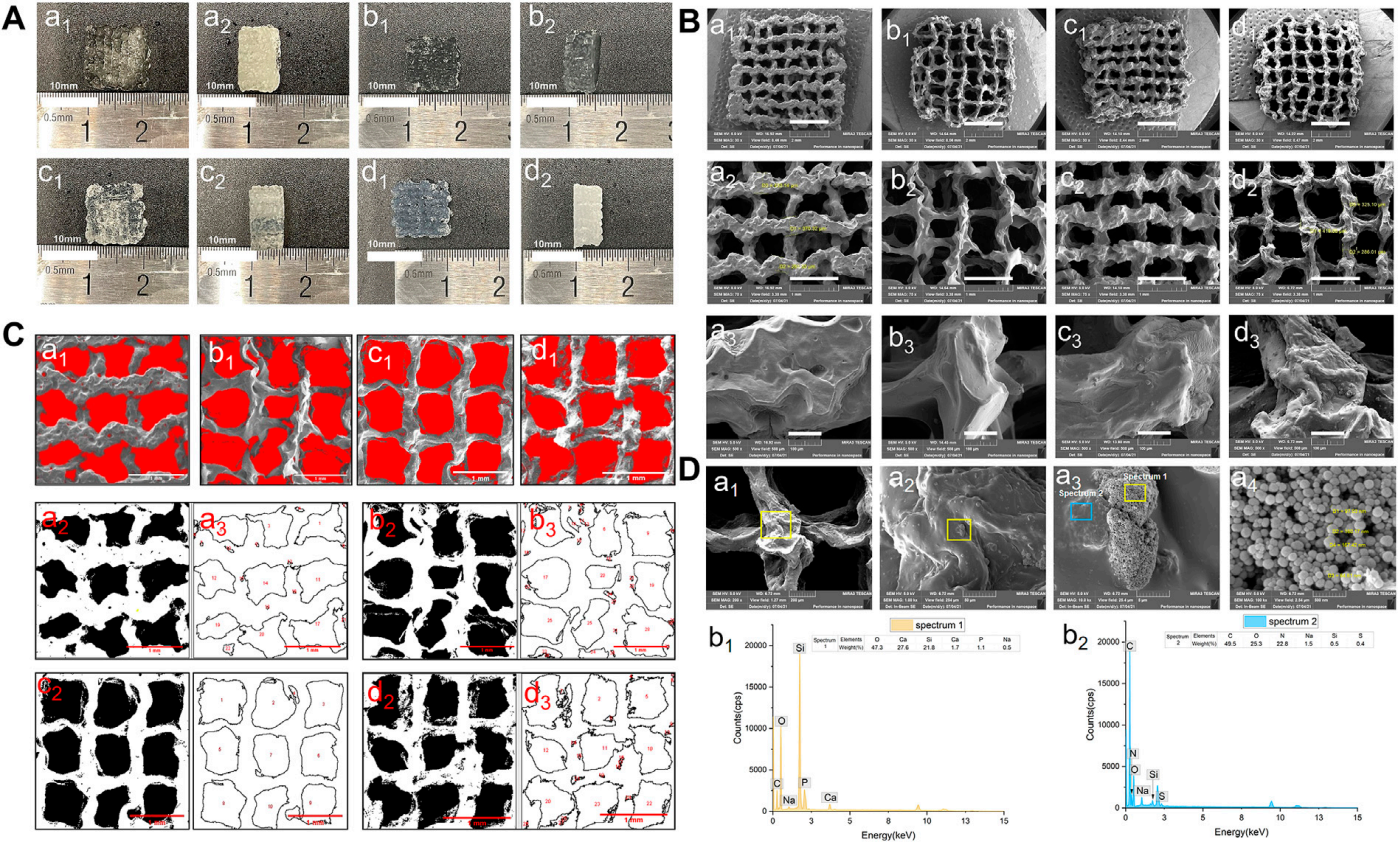
Morphology of 3D-printed mesoporous BG/GelMA macropore scaffolds. **(A)** Top and side-views of the macro morphology. a1–a2) 0% BG group; b1–b2) 1% BG group; c1–c2) 5% BG group; d1–d2) 10% BG group; **(B)** Representative scanning electron microscopic images at 30x, 75x, 500x magnification. a1–a3) 0% BG group; b1–b3) 1% BG group; c1–c3) 5% BG group; d1–d3) 10% BG group; **(C)** and pore sizes of scaffolds. a1–a3) 0% BG group; b1–b3) 1% BG group; c1–c3) 5% BG group; and d1–d3) 10% BG group; Note: all the darkest regions of the image became red; contours of the pores were traced and coded (from a2, a3 to d2,d3); **(D)** The representative SEM images of the 10% BG group scaffolds from a1) 200x; b1) 1.0kx; c1) 10.0kx to d1)100kx and SEM-energy-dispersive X-ray spectroscopy (EDS) analysis of two lumps in a3. b1) graph of EDS analysis of the yellow box area in panel a3; b2) graph of EDS analysis of the blue box area in panel a3.

The lattice scaffold design (7 × 7, meaning 7 columns and 7 rows) with 0◦, 90◦ stacking (12 layers), and 1.2 mm filament space were used to construct the 3D scaffolds with lateral and vertical interconnected pores. Four groups of biomaterial inks were successfully printed into 10.0 mm×10.0 mm×5.0 mm (long×wide×height) 3D porous scaffolds and the macro morphology of the scaffolds were shown in Figure 3A. All groups of BG/GelMA biomaterial inks were printed into integrated and structured scaffolds, while the obvious collapse on the bottom and twisted filaments were observed in the group of biomaterials without BG NPs. With the increase in the concentration of BG NPs, the BG/GelMA scaffolds (1% BG, 5% BG, and 10% BG) became more and more regular and uniform, and were less prone to collapse.

The SEM images of the BG/GelMA scaffolds were shown in Figure 3B. All four scaffolds had uniformly interconnected macropores (Figure 3B), indicating the use of the BA/GelMA biomaterial inks and the extrusion-based 3D printing technique which allowed for precise control of the pore size and structure. Furthermore, the mean pore sizes and porosities of the scaffolds of all groups were analyzed by ImageJ (Figure 3C) and summarized in Table 3. The porosity of the BG/GelMA scaffolds without BG NPs was 43.25%, while it became higher than 50% after the addition of BG NPs. Furthermore, the mean pore size of the BG/GelMA scaffolds increased from ∼300 μm to ∼400 μm by the incorporation of 1%–5% BG NPs, while decreasing to ∼300 μm by the incorporation of 10% BG NPs.

**TABLE 3:** Porosity and pore size analysis of 3D-printed BG/GelMA scaffolds.

Scaffolds		0% BG group	1% BG group	5% BG group	10% BG group
Porosity		43.252%	52.163%	55.03%	52.691%
Pore size (mm)	Mean	0.333	0.424	0.446	0.304
	SD	0.026	0.113	0.065	0.034

By analyzing the SEM images, we observed that the surface of the BG/GelMA scaffolds without BG NPs was the smoothest. With the increase of BG NP content in the scaffold, the scaffolds’ surface became coarser and coarser (Figures 3B,D). At high magnification, spherical nanoparticles embedded in the scaffolds were seen in the 10% BG group, with nanoparticle diameters around 90 nm (Figure 3D). Furthermore, a strong silicon (Si), calcium (Ca) and phosphate (P) peak in the EDS spectra confirmed that the BG NPs were successfully incorporated into the biomimetic BG/GelMA scaffolds by 3D printing (Figure 3D).

**FIGURE 4:**
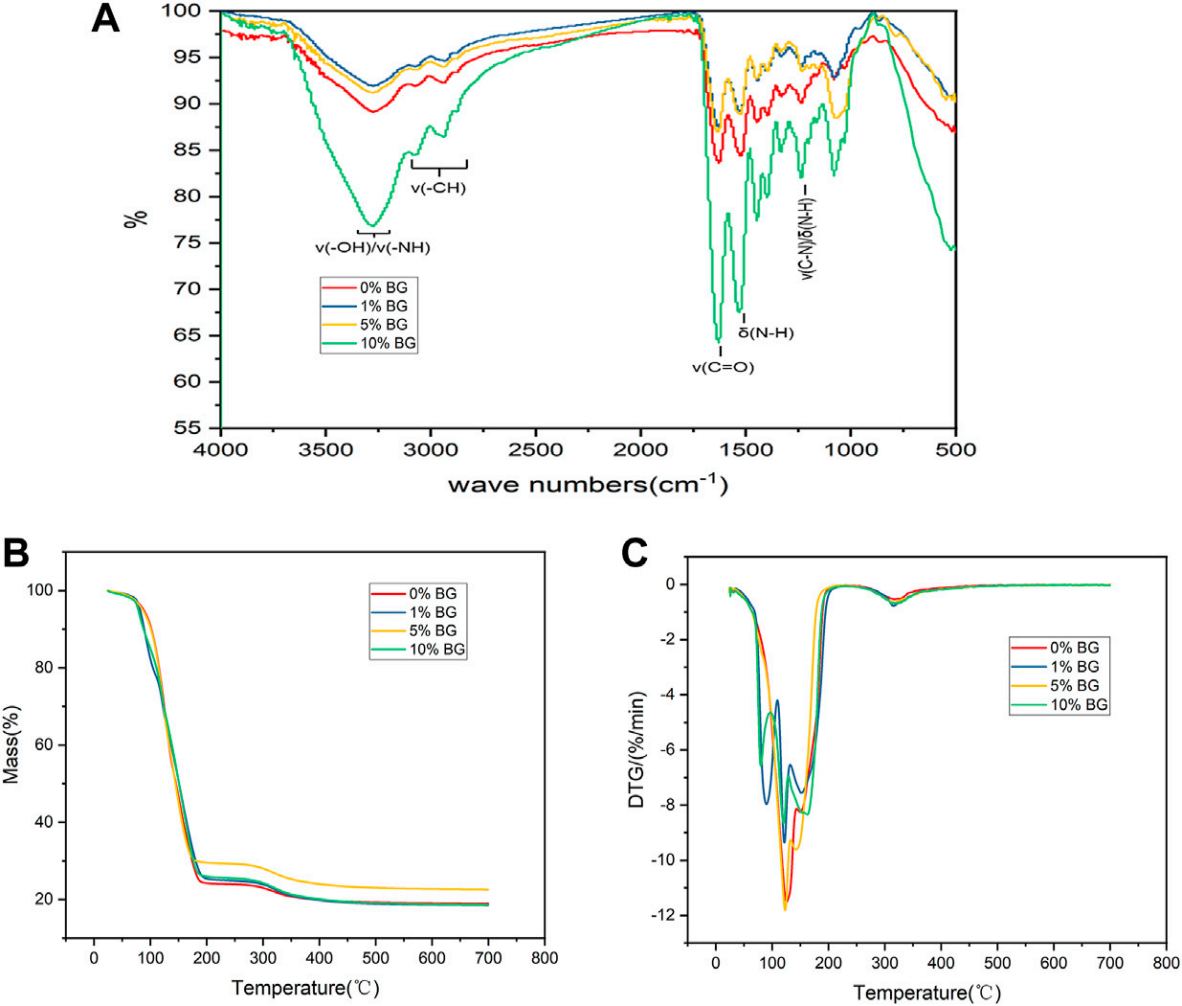
Characterization and thermal properties analysis of 3D-printed mesoporous BG/GelMA macropore scaffolds **(A)** FTIR spectra of BG/GelMA scaffolds with different mass ratios of BG NPs. The symbol ‘ν’ means stretching vibration while δ means bending vibration; **(B)** Thermogravimetric analysis (TGA); **(C)** Derived thermogravimetric (DTG) of scaffolds.

The FTIR spectra of the BG/GelMA scaffolds showed a sharp intense peak at 1640 cm^−1^ corresponding to C=O bonds, and two typical peaks at 1,530 cm^−1^ and 1,240 cm^−1^ related to N–H bending and C–N stretching plus N–H bending, respectively (Figure 4A). Moreover, a broad peak at 3,275 cm^−1^ representing the associated signal for the O-H and N-H groups and a band at 2,940 cm^−1^ representing the C-H stretching groups were also observed. Furthermore, the thermal degradation behavior of the four biomimetic BG/GelMA scaffolds was characterized by using TGA analysis. As shown in Figures 4B, C, the TGA curve showed that all scaffolds began to lose integrity between 80°C and 120°C, probably caused by the loss of water molecules. At temperatures above 150°C, the degradation of GelMA started and the residual mass for scaffold 0%BG, 1% BG, 5% BG, and 10% BG was 18.94%, 18.52%, 22.59%, and 18.61%, respectively, when heating temperature reached 700 °C.

### 3.3 Mechanical and degradational properties of the biomimetic BG/GelMA scaffolds

**FIGURE 5:**
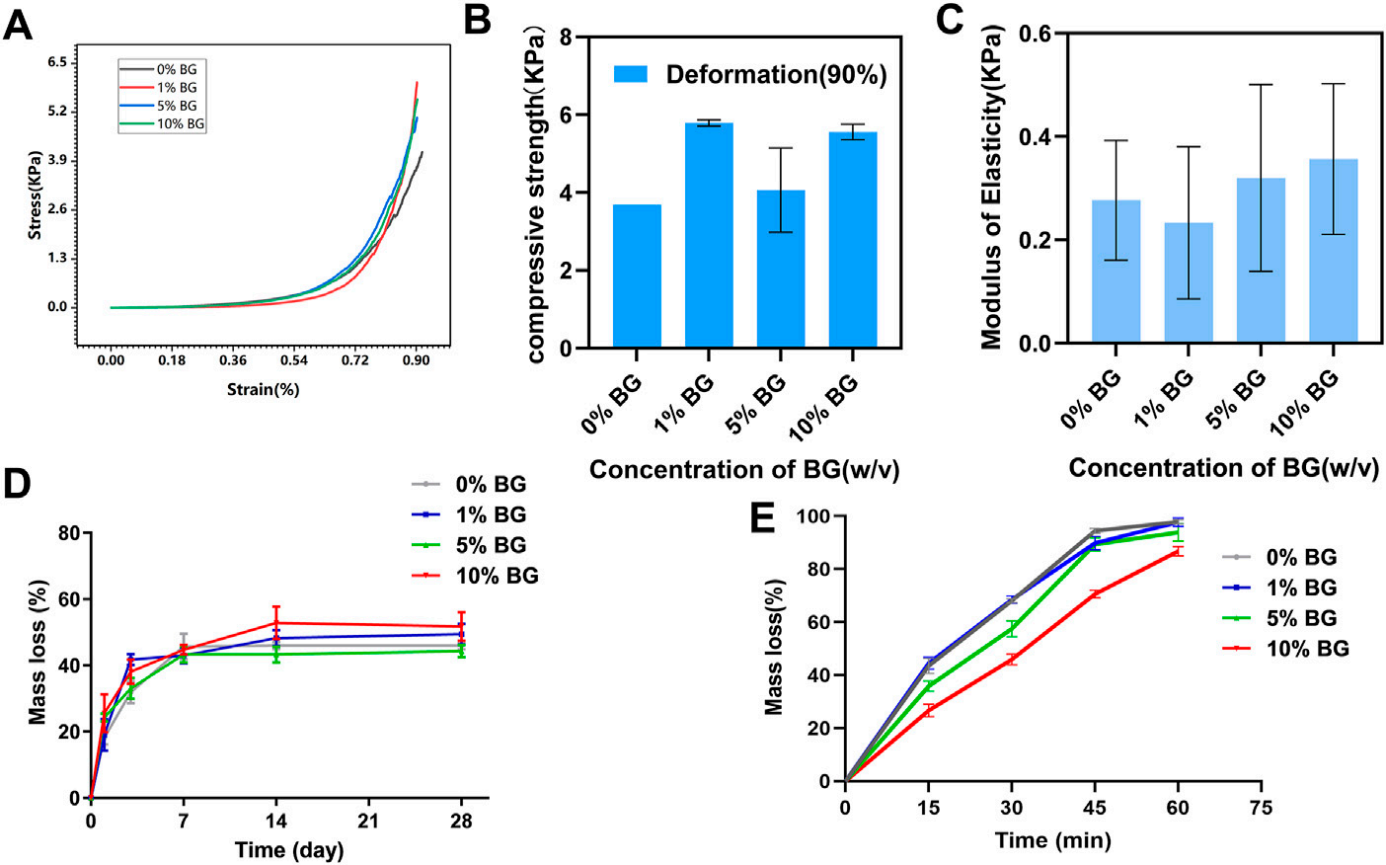
Mechanical and degradation properties of the biomimetic BG/GelMA scaffolds **(A)** Representative compressive stress–strain curve of the biomimetic BG/GelMA scaffolds. Grey: 0% BG group; Red: 1% BG group; Blue: 5% BG group; Green: 10% BG group; **(B)** Compressive strength of the scaffolds at the deformation of 90%; **(C)** Elastic modulus of the scaffolds. N = 3 samples per group; **(D)** *In vitro* degradation in PBS solution at 37°C and **(E)** enzymatic degradation of scaffolds 0% BG, 1% BG, 5% BG, and 10% BG. Grey: 0% BG group; Blue: 1% BG group; Green: 5% BG group; Red: 10% BG group; Data represent the mean ± SD, n = 3.

The compressive mechanical properties of the biomimetic BG/GelMA scaffolds were investigated to verify whether the mechanical properties of tissue engineering scaffold were able to maintain the structural stability when it was cultured *in vitro* or implanted *in vivo* (Wang et al., 2018). From the compressive stress–strain curve (Figure 5A), the trends in scaffolds 0% BG, 1% BG, 5% BG, and 10% BG were consistent and the inflection points were all around 70% of the deformation. The mean compressive strength at 90% deformation of scaffolds 0% BG, 1% BG, 5% BG, and 10% BG were 3.679±0.001, 5.790±0.001, 4.065±0.001, and 5.556±0.001 KPa, respectively, and the moduli of elasticity were 0.277±0.116, 0.233±0.147, 0.320±0.181, and 0.357±0.146 KPa, respectively (Figures 5B,C). There was no significance in compressive strength and modulus elasticity between the scaffolds 0% BG, 1% BG, 5% BG, and 10% BG.

As displayed in Figure 5D, all scaffolds’ mass loss and degradation increased with increase in time. Till 14 days, the curve of mass loss tended to be flat which meant that the degradation of the BG/GelMA scaffolds was brought into a steady state. After degradation for 28 days, the weight loss of all groups of scaffolds tended to be around 50%.

To further evaluate enzymatic degradability, the biomimetic BG/GelMA scaffolds were incubated in collagenase solutions for 15 min, 30 min, 45 min, and 60 min, and the degradation rates of the various scaffolds are shown in Figure 5E. Compared to various scaffolds in PBS, the degradation of four BG/GelMA scaffolds in collagenase solutions became faster and the curve of mass loss became steeper. Notably, after 60 min, the mass loss of scaffolds 0% BG, 1% BG, and 5% BG were all close to 100% except for scaffold 10% BG, which exhibited a slightly slower degradation rate, indicating that the degradation of BG/GelMA scaffolds could be delayed by the incorporation of BG NPs.

### 3.4 Cell adhesion and growth on the surface of the biomimetic BG/GelMA scaffolds

**FIGURE 6:**
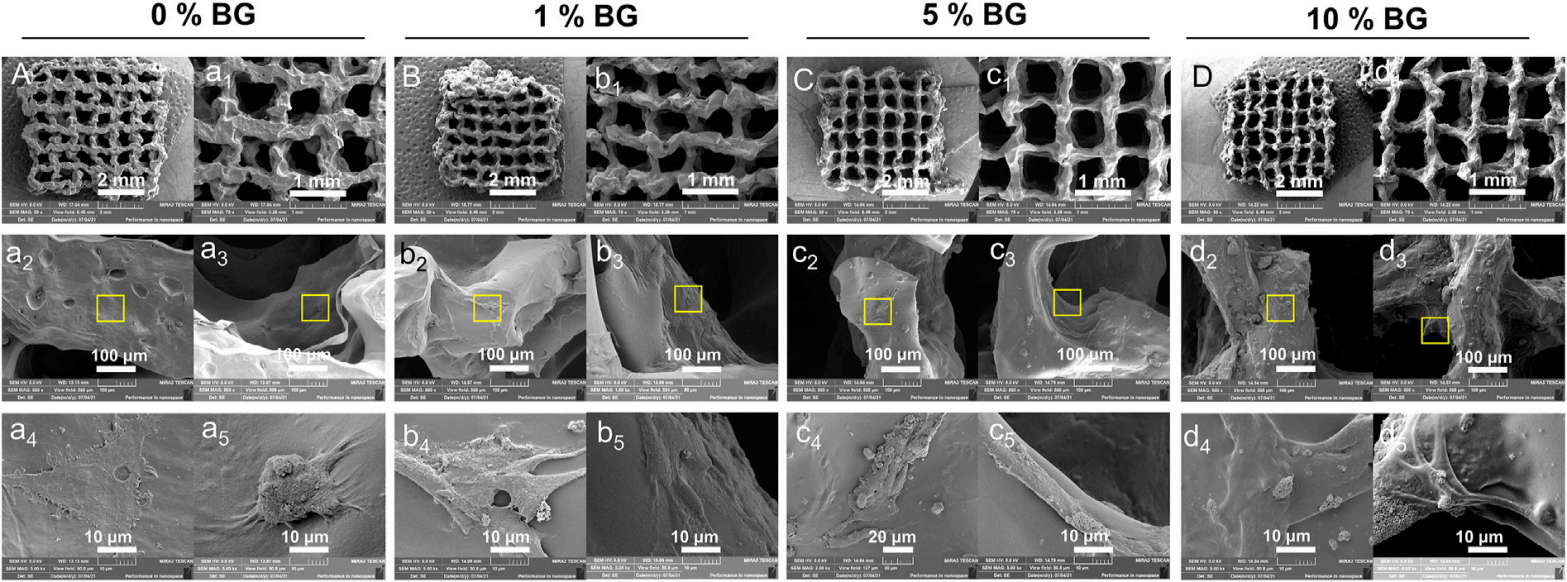
Cell morphology of HPDLCs in 3D-printed biomimetic BG/GelMA macropore scaffolds by SEM. Cells were cultured in scaffolds 0% BG, 1% BG, 5% BG, and 10% BG for 24 h **(A,B,C, and D)** SEM images of integral scaffolds with cells from scaffolds 0% BG, 1% BG, 5% BG, and 10% BG at 30x magnification. (a_1_, b_1_, c_1_, and d_1_) SEM images of partial scaffolds with cells from all groups at 75x magnification. (a_2_, b_2_, c_2_, and d_2_) SEM images of cells in the outer surface and (a_3_, b_3,_ c_3_, and d_3_) inner pore surface of scaffolds at 500x magnification. (a_4-5_, b_4-5_, c_4-5_, and d_4-5_) Magnifying images of cells of the yellow box area at 5.0kx magnification. A/a_1_-a_5_) 0% BG group; B/b_1_-b_5_) 1% BG group; C/c_1_-c_5_) 5% BG group; and D/d_1_-d_5_) 10% BG group.

There were more and more HPDLCs adhering to the surface of scaffolds 0% BG, 1% BG, 5% BG, and 10% BG, and a large proportion of them aggregated around the coarse-faced mass composed of BG NPs under a high-power scanning electron microscope (Figure 6A). According to clumps with rough surfaces formed with inorganic particles at the macro level and the crude surface of BG nanospheres at the micro-level (Figures 3C, D), the adhesion of HPDLCs was promoted. Also, the cells grew flat and spread both on the scaffold surface and the inner region of the pores (Figure 6 a2, a3–d2, d3). BG particles could be seen on and around the cells, and the cells with BG particles around them stretched better (Figure 6 a4, a5–d4, d5).

### 3.5 Osteogenic/cementogenic differentiation of HPDLCs on the biomimetic BG/GelMA scaffolds

**FIGURE 7:**
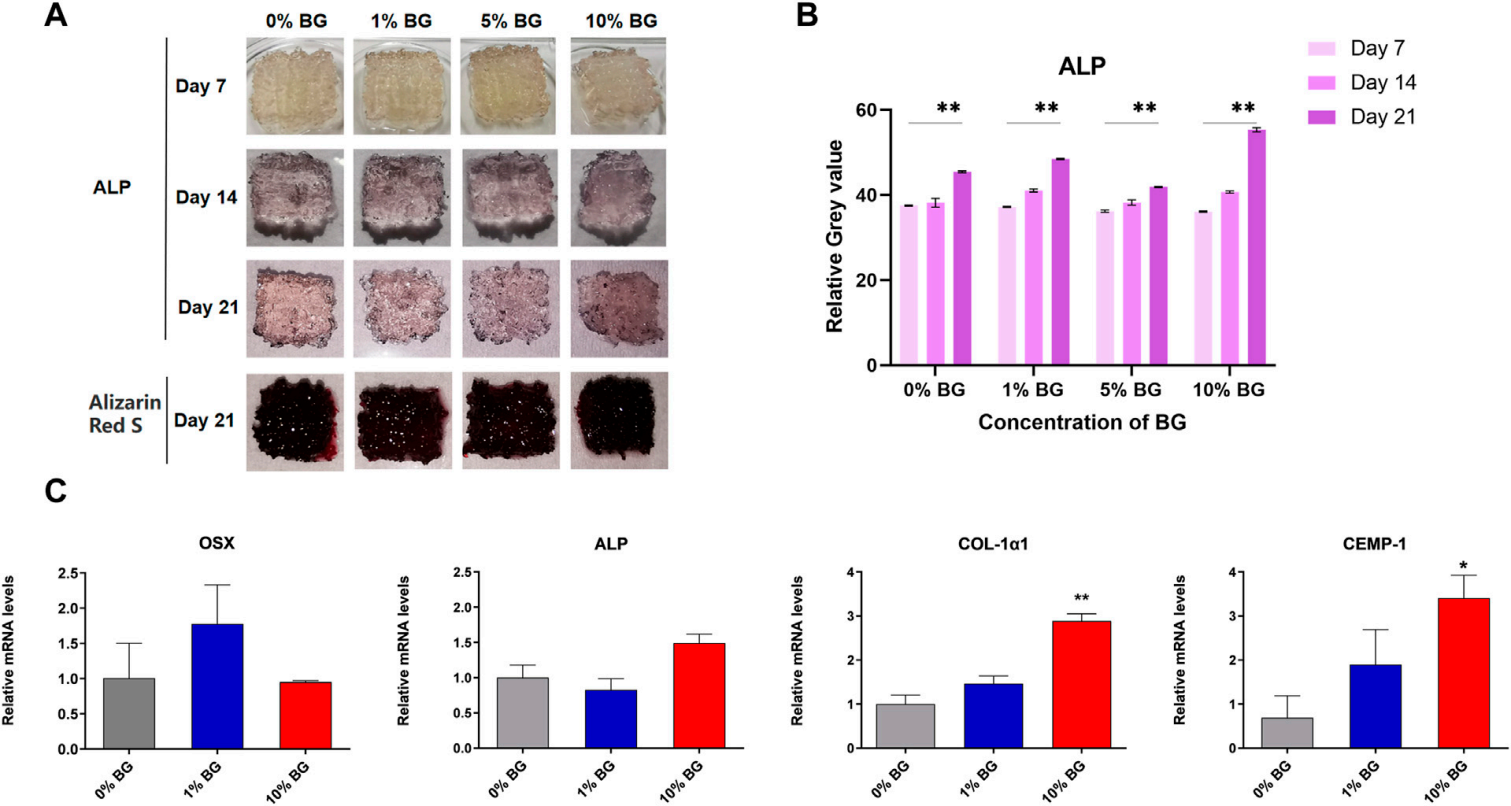
Osteogenic/cementogenic differentiation of HPDLCs on the biomimetic BG/GelMA scaffolds **(A)** Human periodontal ligament cells HPDLCs were cultured in macroporous BG/GelMA scaffolds *in vitro*. Alkaline phosphatase and alizarin red staining were performed every week. Photos of scaffolds on days 7, 14, and 21 were shown. **(B)** The Quantitative analysis of ALP staining. ****p < 0.01. n = 3 for each group. **(C)** The osteogenic and cementogenic quantification and gene expression of HPDLCs seeded on scaffolds 0% BG, 1% BG, and 10% BG for 7 days. n = 3 for each group. **p < 0.01, *p < 0.05.

Alkaline phosphatase (ALP) and Alizarin Red staining results of scaffolds 0% BG, 1% BG, 5% BG, and 10% BG were shown in Figure 7A. It could be seen that the scaffolds of each group cultured with HPDLCs were stained, and the staining deepened with the increase of culture days, which was attributed to enhanced osteogenic differentiation. However, the difference between the scaffolds of different BG concentrations was not obvious under observation of the naked eye.

As shown in Figure 7C, the real-time PCR data showed that the scaffold 10%BG group exhibited the highest expression of osteogenesis and cementogenesis markers (Col-1α1 and CEMP-1) compared to those of scaffolds 0%BG and 1%BG on day 7 (*p* < 0.01). However, the expressions of OSX and ALP showed no significance between scaffolds 0% BG, 1% BG, and 10% BG. We suspected that it might be related to the regulatory mechanisms of different genes, which need further research.

**FIGURE 8:**
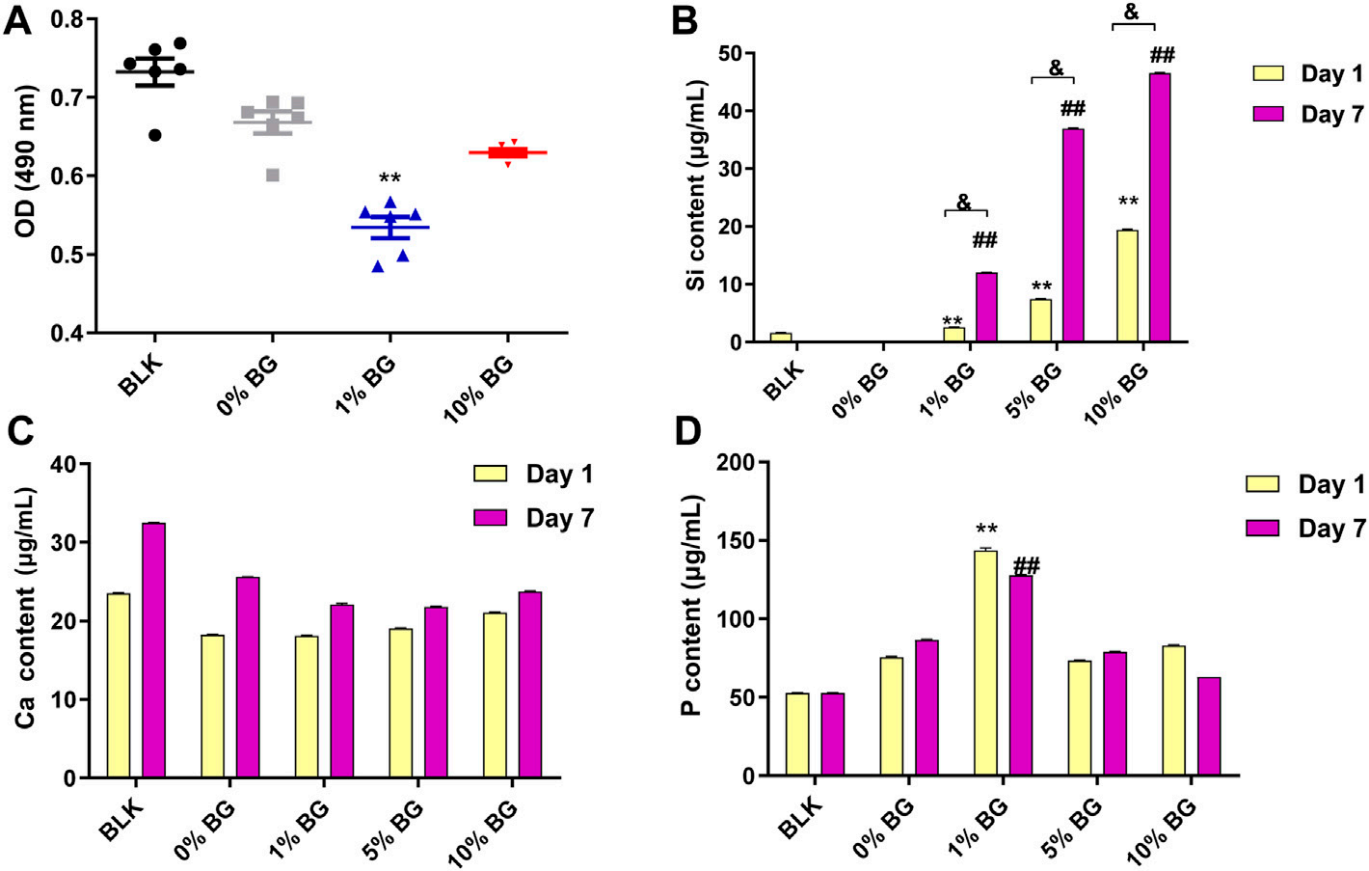
Cell viability of HPDLCs by using MTS assay and the ion release of the biomimetic BG/GelMA scaffolds by ICP-MS **(A)** The OD (490 nm) values in the BLK (Blank: flat plate) group and the scaffolds 0% BG, 1% BG, and 10% BG groups on day 3; The Ca ion release **(B)**, the Si ion release **(C)**, and the P ion release in the BLK group and the scaffold 0% BG, 1% BG, and 10% BG groups on day 1 and day 7. The data represent the mean ± SD, n = 3. **,##,& *p* < 0.01. MTS: (3-(4,5-dimethylthiazol-2-yl)-5-(3carboxymethoxyphenyl)-2-(4-sulfophenyl)-2H-tetrazolium).

The cell viability assay result is shown in Figure 8A. After 3 days of culture, a slight decrease in cell viability in both scaffold 1% BG and 10% BG group was observed compared to the scaffold 0% BG group, and the difference was statistically significant (*p* < 0.01), which revealed that the addition of BG had a negative effect on cell proliferation. However, as the concentration of BG increased, the cell viability was rising rather than falling.

The ion release of the biomimetic BG/GelMA scaffolds with different ratios of BG NPs was investigated by ICP-MS. The amount of silicon ions released from both scaffolds 1% BG and 10% BG was significantly increased in a time-dependent manner (Figure 8B). Scaffolds 10% BG exhibited the highest level of silicon ions release on day 1 and day 7 compared to other scaffolds (*p* < 0.01), which is attributed to the highest concentration of BG NPs. Additionally, in line with the degradation of the scaffolds, the release of silicon ions surged during the first week. No significance was observed in the calcium ion release (Figure 8C). As for phosphorus ions affected by PBS solutions, there is no clear pattern of release (Figure 8D).

## 4 Discussion

In this study, to stimulate endogenous periodontal tissue-regeneration, a novel biomimetic BG/GelMA scaffold with a large pore size and a rough surface was successfully developed by extrusion-based 3D bioprinting. Compared with solvent casting, gas forming, emulsification freeze-drying, and so on, the 3D-printing technique has distinctive advantages in flexibility and accurate control of the macrostructure and pore size (Turnbull et al., 2018). For the purpose of great shape fidelity of extrusion-based 3D-printed scaffolds, the printability of biomaterial inks, referring to the “suitable” extrudability, filament formation, and shape fidelity, is as essential as the design and characterization of biomaterial scaffolds (Gao et al., 2019) (Schwab et al., 2020a). Among biomaterial inks, GelMA shows great potential for extrusion 3D printing, which allows the preparation of hydrogel-based scaffolds with controllable shapes for repairing personalized and irregular periodontal defects. However, the high self-healing ability and the low mechanical strength of GelMA make it difficult for GelMA scaffolds to obtain macroporous structures with good shape fidelity (Liu et al., 2017). To overcome the limits of GelMA-based inks, in this work, the mesoporous BG NPs were incorporated into GelMA to prepare the BG/GelMA biomaterial inks and to improve the shape fidelity.

To investigate whether the mesoporous BG NPs improved the printability of the GelMA biomaterial inks, the critical parameters of rheological properties such as viscoelasticity and shear-thinning were measured. Generally, viscoelasticity displays viscous flow and elastic shape-retention of biomaterial inks, while shear-thinning means their viscosity decreases with increasing shear rate. Compared to the GelMA inks without BG NPs, both clearer line viscoelasticity and typical shear-thinning were observed in the GelMA biomaterials inks after incorporation of BG NPs from 1% to 10% (Figure 2). Our results demonstrated that the as-prepared BG/GelMA inks could be easily extruded from the nozzle due to the good shear-thinning property (Gao et al., 2019). Additionally, Figure 2D showed that the storage modulus (G′) of the BG/GelMA biomaterial inks was higher than the loss modulus (G″) indicating that the incorporation of BG NPs could improve the shape fidelity of 3D-printed scaffolds (Kamkar et al., 2021, 2022).

Next, the stacking lattice scaffold (7×7) with 1.2 mm filament distance and 12 layers with a large porous structure (∼300 μm of pore size) was designed for clinically relevant periodontal defect applications. By extrusion-based 3D printing, the resultant multilayered scaffold was about 10.0 mm×10.0 mm×5.0 mm and the pore size was ranged from 304 μm to 446 μm, which is defined as macropore (Figure 3). The 3D-printed GelMA scaffolds without BG NPs addition showed the bottom had collapsed, indicating poor filament support and stability. With the incorporation of BG NPs (scaffolds 1%BG, 5%BG, and 10%BG), there was less collapse in the scaffolds with higher porosity, suggesting that the addition of mesoporous BG NPs not only increased the printability of biomaterial inks and endowed the scaffolds with a stable macropore structure of good shape fidelity, but also lowered the self-healing degree of GelMA hydrogel inks to ensure the high porosity of scaffolds (Figure 3). Our results were consistent with previous studies showing incorporation of inorganic NPs such as nanoclay, and MXene Nanosheets/Gold NPs into the GelMA solutions could improve the shape fidelity and printability of GelMA inks (Boularaoui et al., 2021).

We further characterized the as-prepared BG/GelMA scaffolds by FTIR spectra. As shown in Figure 4A, the FTIR spectra of the BG/GelMA scaffolds showed typical peaks of gelatin as described for GelMA in previous studies (Rahali et al., 2017). Interestingly, in the GelMA scaffold with 10% BG, the intensity of the O-H groups and N-H amino band was obviously reduced, which is ascribable to the incorporation of BG NPs. Our results were consistent with other studies demonstrating that the incorporation of biphasic calcium phosphate NPs could cause the decrease in peak intensity of the O-H band and N-H amino band of GelMA hydrogels (Choi et al., 2021). Moreover, the thermal stability of the as-prepared BG/GelMA scaffolds was analyzed by using the TGA method. It was observed that the incorporation of BG NPs did not significantly affect the thermal degradation behavior of the biomimetic Gel/MA scaffolds (Figures 4B,C).

It is well-recognized that the composition, structure (e.g. pore size, porosity), and properties (e.g. stiffness, compressive strength, elastic modulus, etc.) of biomaterial scaffolds are critical parameters affecting stem-cell fate and tissue regenerative outcomes (Swanson et al., 2021). An ideal 3D architecture of the scaffold should have an interconnected porous structure with high porosity to decrease the obstacles to the cell and nutrient migration (Turnbull et al., 2018), and the osteoblastic cell adhesion on biomaterials is an important event in initiating and regulating cell survival, migration, recruitment, and osteogenic differentiation (Chen et al., 2018). In this study, compared to the GelMA scaffold without BG NPs (Figure 6 a5), cell attachment and spreading were enhanced on scaffolds with BG incorporation, where cells inside the pore could also stretch (Figure 6 b5-d5). Most of the cells were always found to adhere to BG NP-doped rough surface (Figure 6D). The possible mechanism underlying better cell adhesion and spread in BG groups might be attributed to the rough surface modified by the bioactive glass (Figure 3D) and the highly connected and stable macroporous structure (Figure 3; Table 3). For the rough surface, several studies stated that roughness increased adhesion (Shapira and Halabi, 2009; Boyd et al., 2021). Furthermore, X. Shi et al. found that the topography itself was also important for cell adhesion on a rough protein-resistant surface (Shi et al., 2012). These research studies indicated that the effect of rough surfaces on cell adhesion should not be ignored.

Moreover, the construction of cell-living spaces by macropores in scaffolds is also essential for the establishment of cellular functions, such as cell growth, division, proliferation, and differentiation (Fan and Wang, 2017). The introduction of macroporous structures in scaffolds brings cell-adhesive surfaces and spaces, which is beneficial for cell adhesion, spreading, proliferation, and increased cell–cell contacts (Fan and Wang, 2017). Meanwhile, a porous structure may have offered a better environment for cell proliferation and albumin production through the enhanced mass transfer of nutrients, oxygen, and waste removal, which is essential for cell growth (Hwang et al., 2010) (Fan and Wang, 2015) (Turnbull et al., 2018). A recent study of developing biomaterial scaffolds to maintain the stemness of skeletal bone marrow mesenchymal stem cells (BMSC) gave evidence that different pore sizes had different regulatory effects on stem cell differentiation, where BMSC growing in large pores (>250 μm) have a trajectory toward osteogenic differentiation, while small pores (<125 μm) maintain stemness and prevent the differentiation of BMSC *in vivo* and *in vitro* (Swanson et al., 2021). Consistently, our results also confirmed that large pores greater than 250 μm in diameter could induce osteogenic differentiation of HPDLCs in all 3D-printed GelMA-based scaffold groups (Figure 7).

Although mechanical properties are also important parameters, the mechanical properties of the scaffolds in this study did not significantly improve (Figures 5A–C). Osteogenic and cementogenic differentiations of HPDLCs were demonstrated by alkaline phosphatase (ALP) and Alizarin Red staining as well as up-regulating gene expressions of *Col-1α1*, *ALP*, *CEMP-1*, and *OSX*. ALP has clear functions in the initial stages of cells’ osteoblastic differentiation and growth-plate calcification, which is produced early in growth and is easily found on the surface of the cell and in matrix vesicles of all bones and calcifying cartilages (Vimalraj, 2020). Alizarin Red can chelate with calcium ions to form complexes that can be used to mark calcium nodules formed by osteogenesis differentiation of stem cells (Li et al., 2020) (Gregory et al., 2020). In this study, higher ALP staining of all the groups was remarkably exhibited on the 14^th^ and 21^st^ days compared to that on the 7^th^ day, meaning a trajectory toward osteogenic differentiation from the 14^th^ day (Figure 7A). The same, mineralized nodule formation was observed on the 21^st^ day for all groups. Col-1α1, ALP, and OSX are all osteo-specific genes and are necessary for osteogenic and cementogenic differentiations (Nakashima et al., 2002; Chen et al., 2009). CEMP-1, as a marker protein for cementoblast-related cells, regulates cementogenic differentiation in stem cells (Komaki et al., 2012) (Sanz et al., 2021). Compared to the 3D-printed GelMA scaffold without BG NPs, a significant up-regulation in the gene expressions of Col-1α1 and CEMP-1 was observed in the scaffold 10% BG group, suggesting that the enhanced osteogenic and cementogenic differentiations in HPDLCs were more likely to be related to Si ions release during scaffold degradation (Figure 8B). The most mass loss occurs (Figure 5D) and relatively rapid Si ions release from the scaffold 10% BG group during the first week (Figure 8B). The osteogenic effect induced by Si ions was observed in some previous studies (Odatsu et al., 2015). Silicon ions, as ionic products from bioactive glass degradation, could facilitate the induction of collagen type 1 (Col(I)α1, Col(I)α2) synthesis, and in turn, enhance the expression of downstream markers such as alkaline phosphatase (ALP), Runx2, and osteocalcin (OCN) during osteoblast differentiation (Varanasi et al., 2009; Varanasi et al., 2012; Saffarian Tousi et al., 2013). In a word, BG NPs could contribute to the enhancement of HPDLCs' osteogenic differentiation by releasing inorganic bioactive ions with the help of macroporous structures, rough surface, and GelMA components.

## 5 Conclusion

In summary, we successfully developed a novel mesoporous bioactive glass (BG)/GelMA biomimetic scaffold with a large pore size (∼300 μm) by 3D printing. Our results showed that the incorporation of mesoporous BG NPs significantly improved shape fidelity, surface roughness, and bioactivity of 3D-printed macropore GelMA scaffolds, resulting in the enhanced effects on cell attachment and spreading and promoting osteogenic/cementogenic differentiation in periodontal ligament cells, evidenced by the excellent maintenance of the macropore structure, the visibly improved cell-spreading, the release of bioactive ions (e.g. Si^4+^, Ca^2+^), the up-regulation of gene expressions of osteogenesis and cementogensis, the increase in ALP activity and calcium nodules, suggesting that BG NPs could endow GelMA-based scaffold with excellent structural stability and the ability to promote osteogenic/cementogenic differentiation. Our findings demonstrated the great potential of the newly formulated biomaterial inks and biomimetic BG/GelMA scaffolds for being used in periodontal tissue regeneration and provide important insights into the understanding of cell–scaffold interaction in promoting regeneration of functional periodontal tissues.

## Data Availability

The original contributions presented in the study are included in the article/supplementary material, further inquiries can be directed to the corresponding authors.

## References

[B1] Alge D. L. Anseth K. S. (2013). Lighting the way. Nat. Mat. 12 (11), 950–952. 10.1038/nmat3794 24150411

[B2] Boularaoui S. Shanti A. Lanotte M. Luo S. Bawazir S. Lee S. (2021). Nanocomposite conductive bioinks based on low-concentration GelMA and MXene nanosheets/gold nanoparticles providing enhanced printability of functional skeletal muscle tissues. ACS Biomater. Sci. Eng. 7 (12), 5810–5822. 10.1021/acsbiomaterials.1c01193 34802227 PMC8672345

[B3] Boyd J. D. Stromberg A. J. Miller C. S. Grady M. E. (2021). Biofilm and cell adhesion strength on dental implant surfaces via the laser spallation technique. Dent. Mat. 37 (1), 48–59. 10.1016/j.dental.2020.10.013 PMC7775913 33208265

[B4] Chen S. Gluhak-Heinrich J. Wang Y. H. Wu Y. M. Chuang H. H. Chen L. (2009). Runx2, osx, and dspp in tooth development. J. Dent. Res. 88 (10), 904–909. 10.1177/0022034509342873 19783797 PMC3045537

[B5] Chen S. Guo Y. Liu R. Wu S. Fang J. Huang B. (2018). Tuning surface properties of bone biomaterials to manipulate osteoblastic cell adhesion and the signaling pathways for the enhancement of early osseointegration. Colloids Surfaces B Biointerfaces 164, 58–69. 10.1016/j.colsurfb.2018.01.022 29413621

[B6] Choi J.-B. Kim Y.-K. Byeon S.-M. Park J.-E. Bae T.-S. Jang Y.-S. (2021). Fabrication and characterization of biodegradable gelatin methacrylate/biphasic calcium phosphate composite hydrogel for bone tissue engineering. Nanomater. (Basel, Switz. 11 (3), 617. 10.3390/nano11030617 PMC7999599 33801249

[B7] Cortellini P. Tonetti M. S. (2015). Clinical concepts for regenerative therapy in intrabony defects. Periodontol. 68 (1), 282–307. 10.1111/prd.12048 25867990

[B8] Deliormanlı A. M. Atmaca H. (2018). Biological response of osteoblastic and chondrogenic cells to graphene-containing PCL/bioactive glass bilayered scaffolds for osteochondral tissue engineering applications. Appl. Biochem. Biotechnol. 186 (4), 972–989. 10.1007/s12010-018-2758-7 29797300

[B9] Fan C. Wang D.-A. (2015). Effects of permeability and living space on cell fate and neo-tissue development in hydrogel-based scaffolds: A study with cartilaginous model. Macromol. Biosci. 15 (4), 535–545. 10.1002/mabi.201400453 25557976

[B10] Fan C. Wang D.-A. (2017). Macroporous hydrogel scaffolds for three-dimensional cell culture and tissue engineering. Tissue Eng. Part B Rev. 23 (5), 451–461. 10.1089/ten.TEB.2016.0465 28067115

[B11] Gao J. Ding X. Yu X. Chen X. Zhang X. Cui S. (2021). Cell-free bilayered porous scaffolds for osteochondral regeneration fabricated by continuous 3D-printing using nascent physical hydrogel as ink. Adv. Healthc. Mat. 10 (3), e2001404. 10.1002/adhm.202001404 33225617

[B12] Gao Q. Niu X. Shao L. Zhou L. Lin Z. Sun A. (2019). 3D printing of complex GelMA-based scaffolds with nanoclay. Biofabrication 11 (3), 035006. 10.1088/1758-5090/ab0cf6 30836349

[B13] Gong Jinglei H. Y. Wang J. (2021). Research progress on multiphasic scaffold in periodontal regeneration. Int. J. Stomatol. 48 (5), 563–569. 10.7518/gjkq.2021101

[B14] Gregory C. A. McNeill E. P. Pan S. (2020). “Chapter 2 - preparation of osteogenic matrices from cultured cells,” in Methods in cell biology. Editors Caballero D. Kundu S. C. Reis R. L. (Academic Press), 15–43. 10.1016/bs.mcb.2019.10.009 PMC7444597 32222217

[B15] Grove C. Jerram D. A. (2011). jPOR: An ImageJ macro to quantify total optical porosity from blue-stained thin sections. Comput. Geosciences 37 (11), 1850–1859. 10.1016/j.cageo.2011.03.002

[B16] Hwang C. M. Sant S. Masaeli M. Kachouie N. N. Zamanian B. Lee S.-H. (2010). Fabrication of three-dimensional porous cell-laden hydrogel for tissue engineering. Biofabrication 2 (3), 035003. 10.1088/1758-5082/2/3/035003 20823504 PMC3282162

[B17] Iwata T. Yamato M. Ishikawa I. Ando T. Okano T. (2014). Tissue engineering in periodontal tissue. Anat. Rec. Hob. 297 (1), 16–25. 10.1002/ar.22812 24343910

[B18] Janmaleki M. Liu J. Kamkar M. Azarmanesh M. Sundararaj U. Nezhad A. S. (2020). Role of temperature on bio-printability of gelatin methacryloyl bioink in two-step cross-linking strategy for tissue engineering applications. Biomed. Mat. 16 (1), 015021. 10.1088/1748-605X/abbcc9 33325382

[B19] Jeon J. E. Vaquette C. Klein T. J. Hutmacher D. W. (2014). Perspectives in multiphasic osteochondral tissue engineering. Anat. Rec. Hob. 297 (1), 26–35. 10.1002/ar.22795 24293311

[B20] Kamkar M. Janmaleki M. Erfanian E. Sanati-Nezhad A. Sundararaj U. (2022). Covalently cross-linked hydrogels: Mechanisms of nonlinear viscoelasticity. Can. J. Chem. Eng. n/a. 10.1002/cjce.24388

[B21] Kamkar M. Janmaleki M. Erfanian E. Sanati-Nezhad A. Sundararaj U. (2021). Viscoelastic behavior of covalently crosslinked hydrogels under large shear deformations: An approach to eliminate wall slip. Phys. Fluids 33 (4), 041702. 10.1063/5.0046801

[B22] Komaki M. Iwasaki K. Arzate H. Narayanan A. S. Izumi Y. Morita I. (2012). Cementum protein 1 (CEMP1) induces a cementoblastic phenotype and reduces osteoblastic differentiation in periodontal ligament cells. J. Cell. Physiol. 227 (2), 649–657. 10.1002/jcp.22770 21465469

[B23] Lee C. H. Hajibandeh J. Suzuki T. Fan A. Shang P. Mao J. J. (2014). Three-dimensional printed multiphase scaffolds for regeneration of periodontium complex. Tissue Eng. Part A 20 (7-8), 1342–1351. 10.1089/ten.TEA.2013.0386 24295512 PMC3993023

[B24] Li B. Qin K. Wang B. Liu B. Yu W. Li Z. (2020). Crocin promotes osteogenesis differentiation of bone marrow mesenchymal stem cells. Vitro Cell. Dev. Biol. -Animal. 56 (8), 680–688. 10.1007/s11626-020-00487-w 32935257

[B25] Loh Q. L. Choong C. (2013). Three-dimensional scaffolds for tissue engineering applications: Role of porosity and pore size. Tissue Eng. Part B Rev. 19 (6), 485–502. 10.1089/ten.TEB.2012.0437 23672709 PMC3826579

[B26] Luo Y. Zhang T. Lin X. (2022). 3D printed hydrogel scaffolds with macro pores and interconnected microchannel networks for tissue engineering vascularization. Chem. Eng. J. 430, 132926. 10.1016/j.cej.2021.132926

[B27] Nakashima K. Zhou X. Kunkel G. Zhang Z. Deng J. M. Behringer R. R. (2002). The novel zinc finger-containing transcription factor osterix is required for osteoblast differentiation and bone formation. Cell 108 (1), 17–29. 10.1016/s0092-8674(01)00622-5 11792318

[B28] Odatsu T. Azimaie T. Velten M. F. Vu M. Lyles M. B. Kim H. K. (2015). Human periosteum cell osteogenic differentiation enhanced by ionic silicon release from porous amorphous silica fibrous scaffolds. J. Biomed. Mat. Res. A 103 (8), 2797–2806. 10.1002/jbm.a.35412 PMC6556392 25630903

[B29] Rahali K. Ben Messaoud G. Kahn C. J. F. Sanchez-Gonzalez L. Kaci M. Cleymand F. (2017). Synthesis and characterization of nanofunctionalized gelatin methacrylate hydrogels. Int. J. Mol. Sci. 18 (12), 2675. 10.3390/ijms18122675 PMC5751277 29232870

[B30] Rahaman M. N. Day D. E. Bal B. S. Fu Q. Jung S. B. Bonewald L. F. (2011). Bioactive glass in tissue engineering. Acta Biomater. 7 (6), 2355–2373. 10.1016/j.actbio.2011.03.016 21421084 PMC3085647

[B31] Saffarian Tousi N. Velten M. F. Bishop T. J. Leong K. K. Barkhordar N. S. Marshall G. W. (2013). Combinatorial effect of Si4+, Ca2+, and Mg2+ released from bioactive glasses on osteoblast osteocalcin expression and biomineralization. Mater. Sci. Eng. C 33 (5), 2757–2765. 10.1016/j.msec.2013.02.044 23623093

[B32] Sanz J. L. López-García S. Lozano A. Pecci-Lloret M. P. Llena C. Guerrero-Gironés J. (2021). Microstructural composition, ion release, and bioactive potential of new premixed calcium silicate-based endodontic sealers indicated for warm vertical compaction technique. Clin. Oral Investig. 25 (3), 1451–1462. 10.1007/s00784-020-03453-8 32651645

[B33] Schwab A. Levato R. D'Este M. Piluso S. Eglin D. Malda J. (2020a). Printability and shape fidelity of bioinks in 3D bioprinting. Chem. Rev. 120 (19), 11028–11055. 10.1021/acs.chemrev.0c00084 32856892 PMC7564085

[B34] Schwab A. Levato R. D'Este M. Piluso S. Eglin D. Malda J. (2020b). Printability and shape fidelity of bioinks in 3D bioprinting. Chem. Rev. 120 (19), 11028–11055. 10.1021/acs.chemrev.0c00084 32856892 PMC7564085

[B35] Shapira L. Halabi A. (2009). Behavior of two osteoblast-like cell lines cultured on machined or rough titanium surfaces. Clin. Oral Implants Res. 20 (1), 50–55. 10.1111/j.1600-0501.2008.01594.x 19126108

[B36] Shi X. Wang Y. Li D. Yuan L. Zhou F. Wang Y. (2012). Cell adhesion on a POEGMA-modified topographical surface. Langmuir 28 (49), 17011–17018. 10.1021/la303042d 23157582

[B37] Skallevold H. E. Rokaya D. Khurshid Z. Zafar M. S. (2019). Bioactive glass applications in dentistry. Int. J. Mol. Sci. 20 (23), 5960. 10.3390/ijms20235960 PMC6928922 31783484

[B38] Slots J. (2017). Periodontitis: Facts, fallacies and the future. Periodontol. 75 (1), 7–23. 10.1111/prd.12221 28758294

[B39] Sowmya S. Mony U. Jayachandran P. Reshma S. Kumar R. A. Arzate H. (2017a). Tri-layered nanocomposite hydrogel scaffold for the concurrent regeneration of cementum, periodontal ligament, and alveolar bone. Adv. Healthc. Mat. 6 (7), 1601251. 10.1002/adhm.201601251 28128898

[B40] Sowmya S. Mony U. Jayachandran P. Reshma S. Kumar R. A. Arzate H. (2017b). Tri‐layered nanocomposite hydrogel scaffold for the concurrent regeneration of cementum, periodontal ligament, and alveolar bone. Adv. Healthc. Mat. 6 (7), 1601251. 10.1002/adhm.201601251 28128898

[B41] Sui B. Liu X. Sun J. (2018). Dual-functional dendritic mesoporous bioactive glass nanospheres for calcium influx-mediated specific tumor suppression and controlled drug delivery *in vivo* . ACS Appl. Mat. Interfaces 10 (28), 23548–23559. 10.1021/acsami.8b05616 29947213

[B42] Swanson W. B. Omi M. Zhang Z. Nam H. K. Jung Y. Wang G. (2021). Macropore design of tissue engineering scaffolds regulates mesenchymal stem cell differentiation fate. Biomaterials 272, 120769. 10.1016/j.biomaterials.2021.120769 33798961 PMC8068670

[B43] Tao O. Kort-Mascort J. Lin Y. Pham H. M. Charbonneau A. M. ElKashty O. A. (2019). The applications of 3D printing for craniofacial tissue engineering. Micromachines 10 (7), 480. 10.3390/mi10070480 PMC6680740 31319522

[B44] Tavares M. T. Gaspar V. M. Monteiro M. V. S Farinha J. P. Baleizão C. Mano J. F. (2021). GelMA/bioactive silica nanocomposite bioinks for stem cell osteogenic differentiation. Biofabrication 13 (3), 035012. 10.1088/1758-5090/abdc86 33455952

[B45] Turnbull G. Clarke J. Picard F. Riches P. Jia L. Han F. (2018). 3D bioactive composite scaffolds for bone tissue engineering. Bioact. Mater. 3 (3), 278–314. 10.1016/j.bioactmat.2017.10.001 29744467 PMC5935790

[B46] van Haaften E. E. Duijvelshoff R. Ippel B. D. Söntjens S. H. M. van Houtem M. H. C. J. Janssen H. M. (2019). The degradation and performance of electrospun supramolecular vascular scaffolds examined upon *in vitro* enzymatic exposure. Acta Biomater. 92, 48–59. 10.1016/j.actbio.2019.05.037 31108258

[B47] Varanasi V. G. Leong K. K. Dominia L. M. Jue S. M. Loomer P. M. Marshall G. W. (2012). Si and Ca individually and combinatorially target enhanced MC3T3-E1 subclone 4 early osteogenic marker expression. J. Oral Implantol. 38 (4), 325–336. 10.1563/AAID-JOI-D-11-00108 22913306 PMC6597170

[B48] Varanasi V. G. Saiz E. Loomer P. M. Ancheta B. Uritani N. Ho S. P. (2009). Enhanced osteocalcin expression by osteoblast-like cells (MC3T3-E1) exposed to bioactive coating glass (SiO2-CaO-P2O5-MgO-K2O-Na2O system) ions. Acta Biomater. 5 (9), 3536–3547. 10.1016/j.actbio.2009.05.035 19497391 PMC6568011

[B49] Vimalraj S. (2020). Alkaline phosphatase: Structure, expression and its function in bone mineralization. Gene 754, 144855. 10.1016/j.gene.2020.144855 32522695

[B50] Wang W. Nie W. Liu D. Du H. Zhou X. Chen L. (2018). Macroporous nanofibrous vascular scaffold with improved biodegradability and smooth muscle cells infiltration prepared by dual phase separation technique. Int. J. Nanomedicine 13, 7003–7018. 10.2147/IJN.S183463 30464455 PMC6219111

[B51] Wang Z. Wang Z. Lu W. W. Zhen W. Yang D. Peng S. (2017). Novel biomaterial strategies for controlled growth factor delivery for biomedical applications. NPG Asia Mat. 9 (10), e435. 10.1038/am.2017.171

[B52] Xiao S. Zhao T. Wang J. Wang C. Du J. Ying L. (2019). Gelatin methacrylate (GelMA)-Based hydrogels for cell transplantation: An effective strategy for tissue engineering. Stem Cell Rev. Rep. 15 (5), 664–679. 10.1007/s12015-019-09893-4 31154619

[B53] Yousefi A.-M. Hoque M. E. Prasad R. G. S. V. Uth N. (2015). Current strategies in multiphasic scaffold design for osteochondral tissue engineering: A review. J. Biomed. Mat. Res. A 103 (7), 2460–2481. 10.1002/jbm.a.35356 25345589

[B54] Yue K. Trujillo-de Santiago G. Alvarez M. M. Tamayol A. Annabi N. Khademhosseini A. (2015). Synthesis, properties, and biomedical applications of gelatin methacryloyl (GelMA) hydrogels. Biomaterials 73, 254–271. 10.1016/j.biomaterials.2015.08.045 26414409 PMC4610009

